# Prevalence and factors associated with sleep disorders among children with cerebral palsy in Uganda; a cross-sectional study

**DOI:** 10.1186/s12887-018-0996-z

**Published:** 2018-02-05

**Authors:** Kisughu Munyumu, Richard Idro, Catherine Abbo, Mark Kaddumukasa, Elly Katabira, Ezekiel Mupere, Angelina Kakooza-Mwesige

**Affiliations:** 1 Department of Pediatrics, School of Medicine, Makerere University College of Health Sciences, P.O. Box 7072, Kampala, Uganda; 2 Department of Psychiatry, School of Medicine, Makerere University College of Health Sciences, P.O. Box 7072, Kampala, Uganda; 3 Department of Medicine, School of Medicine, Makerere University College of Health Sciences, P.O. Box 7072, Kampala, Uganda

**Keywords:** sleep disorders, Sleep Disturbance Scale for Children (SDSC), cerebral palsy, children, Uganda

## Abstract

**Background:**

Sleep plays a prominent role in the growth and development of children. Children with cerebral palsy (CP) are more prone to sleep disorders (SDs) than their peers. Children with CP, have a higher prevalence of disorders involving; initiation and maintenance of sleep, sleep-wake transition, excessive sleepiness and arousal. These sleep disorders impact on the quality of life of these children. Despite, having a high prevalence of CP in Uganda, there is a paucity of data that focuses on sleep disorders in CP, including a lack of prevalence estimates of sleep breathing disorder (SBD) in CP. Understanding the prevalence and disorders of sleep within this population would help advise on the development of tailored interventions to address the needs of these children and improve their quality of life. This study determined the prevalence and associated factors of sleep disorders among children aged 2 – 12 years with cerebral palsy in Uganda.

**Methods:**

This was a cross sectional study. All participants had a physical examination and screening with the Sleep Disturbances Scale for Children (SDSC) questionnaire to determine the prevalence of sleeps disorders. A total score (TS) ≥ 51 on the Sleep Disturbances Scale for Children was regarded as abnormal.

**Results:**

A total of 135 participants were recruited. The prevalence of sleep disorders was 43/135 (32%) with 95% CI: (24.0-39.7). The most common type of sleep disorders was a disorder of initiating and maintaining sleep 37(27%). The factors associated with sleep disorders among children with cerebral palsy were bilateral spasticity (*p* = 0.004); OR:(95%CI), 11.193: (2.1 – 59.0), lowest levels of gross motor function V (*p* = < 0.001); OR:(95%CI), 13.182: (3.7 – 47.0) or IV (*p* = 0.007); OR:(95%CI), 12.921: (2.0 – 82.3), lowest level of manual ability V *(p =* 0.004); OR:(95%CI), 11.162: (2.2 – 56.4) and presence of epilepsy (*p* = 0.011); OR:(95%CI), 3.865: (1.4 – 10.9).

**Conclusions:**

The prevalence of sleep disorders among children with cerebral palsy in Uganda is high. Severe disability and presence of epilepsy were associated with sleep disorders among children with cerebral palsy.

## Background

Childhood disability affects millions of children around the world with majority in low- and middle-income countries [1]. Cerebral palsy, one of the leading causes of disability, is a common and serious chronic motor disability, beginning in early childhood and persists throughout the lifespan [2]. Children with cerebral palsy experience sleep disturbances. Sleep is vital for a child’s normal physical growth and psychological health and plays a critical role in the neurological development. Unresolved sleep disturbances which exist for many months place a heavy burden on the family and disrupt normal family life. A study done by Newman et al. of 173 school age children with cerebral palsy attending the Central Remedial Clinic in Dublin, Ireland, found that 39 (22.3%) had a pathological total sleep score [3].Sleep disturbance in patients with cerebral palsy may increase morbidity. Karatas et al. reviewed the records on the deaths of 177 cerebral palsy and found that 19(10.7%) of them were discovered dead during sleep (DDDS) at home [4]. Children with cerebral palsy may have multiple risk factors for sleep disturbance because of the nature of their primary brain injury [5].Various factors contributing to sleep disorders have been proposed including mental retardation [6],visual impairment [7],seizures [8],anti-epileptic medications [9],obstructive sleep apnea [10],restricted movements due to contractures, spasticity and motor impairment [3],pain [11]due to spasticity, dental caries, use of orthoses, etc. Sleep disturbances in patients with cerebral palsy may increase morbidity and mortality. However, data is lacking regarding sleep disorders among children with CP in Uganda to enable development of treatment strategies or interventions. We therefore determined the prevalence and associated factors of sleep disorders among children aged 2 – 12 years with cerebral palsy in Uganda.

## Methods

### Study design

This was a cross-sectional study.

### Setting

The study was conducted in the paediatric neurology clinic at Mulago hospital in Kampala, Uganda. Mulago hospital is a public hospital located 2 km from the city center and serves as a National Referral for the entire country and a general hospital as well as Health Center IV, III for the Kampala metropolitan area (Uganda’s capital city) with an official bed capacity of 1790. It also serves as a teaching hospital for Makerere University College of Health Sciences. The paediatric neurology clinic is under the Department of Paediatrics and Child Health and is run as an outpatient specialized clinic which caters to children with neurological disorders once a week every Thursday between 8 am – 3 pm. It serves as a referral outpatient clinic for the neurological cases from all over the country. Annually the clinic sees about 300 new patients and on each clinic day 25 – 40 children with ages ranging from 2 months to 18 years are attended to; the clinic’s upper age limit is 16 but there are older patients who have not yet been transferred to the adult clinic. In the pediatric neurology clinic, the children are assessed by the team to confirm the diagnosis of CP but also assessed further for any co morbidities through comprehensive history taking, physical examination and also appropriate investigations are carried out to confirm the diagnosis. The pediatric neurology clinic works with the Cerebral Palsy Rehabilitation clinic where physiotherapy/physical therapy treatment modalities and rehabilitation for children with CP are conducted as well as training the caretakers.

### Study period

The study was carried out over a 6 months’ period from June to December 2015.

### Study participants

Participants were children aged 2 - 12 years old with a diagnosis of cerebral palsy attending the two clinics during the study period. Participants were required to have been accompanied by a caregiver who was responsible for providing most of the material and emotional requirements to the child for a period of at least 6 months. Children who met the study inclusion criteria were enrolled into the study. The study inclusion criteria included: Children aged 2-12 years with cerebral palsy attending Mulago hospital general paediatric neurology clinic or the cerebral palsy rehabilitation clinic during the study period.

We excluded severely ill children participants with chronic health problems (bronchial asthma, renal, hepatic, cardiac impairment and known cases of symptomatic paediatric HIV/AIDS) or participants whose caregivers were unable to provide adequate information about the child.

### Sample size calculations

A total sample size of 135 participants was estimated using the Kish Leslie (1965) formula for finite populations, based on a prevalence of sleep disorders in children with cerebral palsy of 22.5% by Newman CJ et al. [3] The formula for the sample size of surveys i.e. the Kish Leslie (1965) formula below was used (adjusted for available population): sample size = n/1 + n/N, where *n* = z^2^ x p (1-p)/e^2^.

### Study procedures

Data was collected using interviewer administered pretested questionnaires for children with Cerebral palsy. The caregivers who presented at the Cerebral Palsy rehabilitation clinic or the Paediatric Neurology clinic were informed about the study, its importance and its objectives and screened for eligibility. Caregivers of eligible children were asked for consent to participate in the study. Participants with consenting parents had a history and a full physical exam performed. The assessments included the Gross Motor Function Classification System (GMFCS) levels, Manual Ability Classification System (MACS) and Communication Function Classification System (CFCS). All participants had screening with the Sleep Disturbances Scale for children (SDSC) questionnaire. Participants were then stratified according to total SDSC score. A total score (TS) ≥ 51 on the Sleep Disturbances Scale for Children (SDSC) was regarded as sleep disorders. The SDSC has been widely used assessing sleep disorders related to cerebral palsy. The questionnaire considers symptom as pertaining to the past 6 months of the child’s life. The internal consistency is high in controls (0.79) and remains at a satisfactory level in sleep disturbances subjects (0.71); the test/pretest reliability is adequate for the total *(I.* =0.71).

The Sleep Disturbance Scale for Children (SDSC) is a 26-item instrument for evaluating sleep.

### Statistical analysis

Data was entered using EPI DATA version 3.1 and exported into STATA version 12 for analysis. Continuous variables were analysed using means, median and standard deviations while categorical variables were analysed using frequencies, proportions and percentages. The prevalence of sleep disorders was determined by obtaining the proportion of children with pathological total SDSC scores among the study participants. Participants were then stratified according to total SCDC score and bivariate analysis conducted. Chi-square or Fisher’s exact test was used for categorical variables. For multivariable analysis, factors with a *p*-value less than 0.2 were entered in the logistic regression analysis. The Students t test was used to compare means (SD) of data which were normally distributed and Mann-Whitney U test was used to compare medians (IQR) for skewed data. *P-*values less than 0.05 were considered significant.

## Results

One hundred thirty-five participants were recruited into the study of whom 33% (45/135) were new referrals to the CP clinic while 67% (90/135) regularly attending the clinics for their attendant follow up care. The majority of participants 69% (93/135) were Baganda who are the largest tribe in Uganda. The gender distribution was similar with males contributing 49% (66/135). The mean age (±SD) of the participants was 3.5(2.0) years. Sixty-six percent (89/135) of the study participants were aged between 2 and 3.99 years. See Table 1.

**Table 1:** Baseline demographic characteristics among the study participants

	Frequency	Percentage (%)
*First visiting hospital*		
Yes	45	33
No	90	67
*Tribe*		
Baganda	93	69
Others	42	31
*Age group of the child(in years)*		
2- < 4	89	66
4- < 6	23	17
6-12	23	17
*Sex*		
Male	66	49
Female	69	51
*Caregiver education level*		
No schooling/primary	51	38
O level/A level/Higher	84	62

Sixty-eight percent (92/135) of the study participants had a normal sleep, while 32% (43/135) children had a total score (TS) ≥ 51 with 95% CI: (24.0-39.7) on the Sleep Disturbance Scale for Children. Six children (4%) had disorder of arousal. The most common type of sleep disturbances was Disorder in initiating and maintaining sleep (DIMS), See Table 2.

**Table 2:** Pathological total scores and types of sleep disorders according to sleep disturbance scale for children (SDSC)

	Disorder of initiating and maintaining of sleep (DIMS)	Sleep-Wake Transition Disorder (WTD)	Sleep hyperhidrosis (SHY)	Sleep Breathing Disorder (SBD)	Disorder of Excessive Somnolence (DOES)	Disorder of Arousal (DA)	Total Score (TS)	95% Confidence Interval (CI)
Score range	7-35	6-30	2-10	3-15	5-25	3-15	26-130	
Mean ± SD	12.5 ± 7.5	8.2 ± 4.0	2.9 ± 2.3	3.8 ± 2.1	6.4 ± 2.9	3.2 ± 0.92	37.24 ± 15.6	
Cut off	≥ 17	≥ 14	≥ 7	≥ 7	≥ 13	≥ 6	≥ 51	
Normal sleep *n* (%)	98 (73)	117 (87)	121 (90)	122 (90)	124 (92)	129 (96)	92 (68)	60.3-76.0
Pathological sleep *n* (%)	37 (27)	18 (13)	14 (10)	13 (10)	11 (8)	6 (4)	43 (32)	24.0-39.7

We determined the factors associated with sleep disorders in patients with cerebral palsy using the biomedical factors such as type and severity of Cerebral Palsy, level of function on the manual ability, gross motor function and communication function scales, presence of epilepsy, caretaker characteristics and relationship with the child and bed sharing at home with the diagnosis of sleep disorders. Markers of a more severely disabled child (bilateral spastic cerebral palsy or poorer scores on the Gross motor function classification scale, communication and manual ability scales) on assessment and epilepsy were the most important risk factors associated with sleep disorders on bivariate analysis, See Tables 3 and 4.

**Table 3:** Bivariate analysis for child bio-medical factors (types of CP, MAC, GMFC, CFC and epilepsy) associated with pathological total scores of sleep disturbances

Variables		Sleep disorders		OR (95% CI)	*p*-value	Chi square *p*-value
		Yes (%)	No (%)			
Types of CP	Bilateral spastic CP	25(58)	36(39)	3.299(1.0-10.9)	0.05	0.152
	Unilateral spastic CP	6(14)	19(21)	1.5(0.4-6.2)	0.575	
	Dyskinetic CP	8(19)	18(20)	2.111(0.5-8.2)	0.282	
	Ataxic CP/non classified	4(9)	19(21)	1		
Gross Motor Function Classification (GMFC)	level I	4(9)	22(24)	1		0.011
	level II	5(12)	20(22)	1.375(0.3-5.8)	0.666	
	level III	2(5)	11(12)	1.000(0.2-6.3)	1	
	level IV	10(23)	16(17)	3.438(0.9-13.0)	0.068	
	level V	22(52)	23(25)	5.261(1.6-17.7)	0.007	
Manual Ability Classification (MAC)	level I	9(21)	33(36)	1		0.007
	level II	4(9)	16(17)	0.917(0.2-3.4)	0.897	
	level III	0(0)	4(4)	1		
	level IV	5(12)	15(16)	1.222(0.3-4.3)	0.753	
	level V	25(58)	24(26)	3.819(1.5-9.6)	0.005	
Communication Function Classification (CFC)	level I	4(9)	27(29)	1		< 0.001
	level II	4(9)	17(18)	1.588(0.3-7.2)	0.549	
	level III	3(7)	13(14)	1.558(0.3-8.0)	0.596	
	level IV	7(16)	18(20)	2.625(0.7-10.3)	0.166	
	level V	25(58)	17(18)	9.926(2.9-33.5)	< 0.001	
Epilepsy	Yes	19(44)	26(28)	2.010(0.9-4.3)	0.07	0.067
	No	24(56)	66(72)	1

**Table 4:** Bivariate analysis for antiepileptic drugs (AED) and social caregiver factors associated with pathological total scores of sleep disturbances

Variables		Sleep disorders		OR (95% CI)	*p*-value	Chi square *p*-value
		Yes (%)	No (%)			
Antiepileptic drugs (AED)	Sodium-valproate	13(68)	15(58)	1		0.638
	Carbamazepine	6(32)	9(35)	0.769(0.2-2.7)	0.686	
	Phenytoin	0(0)	1(4)	–		
	Phenobarbitone	0(0)	1(4)	–		
Child educational level	No-school	43(10)	87(95)			0.119
	Nursery-school	0(0)	5(5)			
Caregiver educational level	No schooling/primary	15(35)	36(39)	1		0.635
	O level/A level/Higher	28(65)	56(61)	1.2(0.6-2.6)	0.636	
Relationship with the child	Mother	34(79)	78(85)	1		0.411
	Other	9(21)	14(15)	1.475(0.6-3.7)	0.413	
Bed sharing all night more than 6 months	Yes	29(67)	71(77)	0.613(0.3-1.4)	0.231	0.229
	No	14(33)	21(23)	1		

All factors in Tables 3 and 4 with a *p*-value < 0.2 on bivariate analysis were entered in a logistic regression model to determine risk factors independently associated with sleep disorders. Features associated with a more severe disability in the child with cerebral palsy and epilepsy were independently associated with a diagnosis of sleep disorders as shown in Table 5. On multivariable model, bilateral spastic cerebral palsy (*p* = 0.004); OR:(95%CI), 11.193: (2.1 – 59.0),gross motor function limitation level V (*p* = < 0.001); OR:(95%CI), 13.182: (3.7 – 47.0),level IV (*p* = 0.007); OR:(95%CI), 12.921: (2.0 – 82.3), manual ability level V *(p =* 0.004); OR:(95%CI), 11.162: (2.2 – 56.4) and epilepsy (*p* = 0.011); OR:(95%CI), 3.865: (1.4 – 10.9) were independently significant factors associated with sleep disorders. Children with bilateral spastic cerebral palsy were 6.4 times more likely to have sleep disorders. Bed sharing was not associated with sleep disorders.

**Table 5:** Multivariate model analysis for factors associated pathological total scores of sleep disorders

Sleep disorders		OR	95% CI	*p*-value
Types of CP	Bilateral spastic CP	11.193	2.1-59.0	0.004
	Unilateral spastic CP	7.725	1.2-51.3	0.034
	Dyskinetic CP	3.570	0.7-18.8	0.134
	Ataxic CP/non classified	1		
Gross motor function	level I	1		
	level II	5.714	1.0-31.3	0.045
	Level III	1.000		
	level IV	12.921	2.0-82.3	0.007
	level V	13.182	3.7-47.0	< 0.001
Manual ability	level I	1		
	level II	2.338	0.4-12.5	0.322
	level III	1.546	0.2-12.4	0.681
	level IV	4.509	0.9-22.5	0.066
	level V	11.162	2.2-56.4	0.004
Relationship with the child	Mother	1		
	Other	2.300	0.7-7.3	0.159
Bed sharing all night more than 6 months	Yes	1		
	No	2.130	0.8-5.8	0.138
Epilepsy	Yes	3.865	1.4-10.9	0.011
	No	1		

## Discussion

### Prevalence of sleep disorders in cerebral palsy

This study set out to determine the prevalence and factors associated with sleep disorders among children with cerebral palsy in Uganda. The study found that, one-third of children with cerebral palsy attending Mulago have sleep disorders. Sleep disorders among this population were associated with severe gross or fine motor function level involvement or disability. This high prevalence of sleep disorders reported at 32%, is higher than what has been described in earlier studies such as Malaysia and several European countries, where the reported prevalence is between 10 and 25% [12]. In Italy, the prevalence was reported as 13% [13] while in Ireland, a prevalence of 22.5% was reported [3]. This might be due the differences in the patient populations, as patients in Mulago had more severe cerebral palsy compared to the other countries. Mulago which serves as a national referral tends to receive more seriously ill patients compared to other hospitals and this can influence the results.

The most common type of sleep disorders was Disorder in Initiating and Maintaining Sleep (DIMS) 27%. Others were Sleep-Wake Transition Disorder (SWTD) 13%, sleep hyperhydrosis 10%, Sleep Breathing Disorder (SBD) 10%, Disorder of Excessive Somnolence (DOES) 8% and then Disorder of arousal(DA) 4%. A similar pattern was found also in both the Malaysian and other studies mentioned above [3, 12, 14]. Several co-morbid problems may affect the initiation of sleep including posture (increased risk of painful reflux), concurrent breathing problems, pain and constipation which are all common in children with CP.

### Factors associated with sleep disorders among children with cerebral palsy

In this study, we found that bilateral, gross motor classification of level V or IV, manual ability of level V and presence of epilepsy were associated with sleep disorders. These patients had more severe functional motor limitation often characterized by bilateral spasticity, experiencing stiffness and contractures suggesting that severe disability is associated with sleep difficulties. Indeed, in the Italians study by ‘Romeo et al., 48% of children with level V on the GMFCS and work reported by Sandella et al., shows that GMFCS predicted sleep problems [13, 15]. The 10% prevalence of hyperhidrosis in the present cohort, a marker of autonomic involvement also suggests a more severe injury and disease.

In this study, presence of epilepsy was associated with sleep disorders. In addition, we did not find an association between social factors such bed sharing and or caregiver factors such as level of educational attainment or relationship with the child (the caretaker being the biological mother or not) were not associated with sleep disorders among our participants similar to findings in Malaysia [12]. Nevertheless, Newman et al. found that bed-sharing was associated with an increase in sleep disorders [3]. This differences is probably due to different socio-cultural perceptions [16]. Further studies are needed to explore the issue of comorbid problems in our settings.

## Conclusions

Approximately one third of children with cerebral palsy have disorders of sleep in a cohort at a national referral and teaching hospital in Uganda. The most common type of sleep disorders in children with cerebral palsy was disorders in the initiation and maintenance of sleep (DIMS).

Severe disability and presence of epilepsy were associated with Sleep disorders among children with cerebral palsy.

## Abbreviations

CFCS: Communication Function Classification System
CP: Cerebral Palsy
DA: Disorder of Arousal
DIMS: Disorder of Initiating and Maintaining of sleep
DOES: Disorder of Excessive Somnolence
GMFCS: Motor Function Classification System
MACS: Manual Abilities Classification System
SBD: Sleep Breathing Disorder
SD: Standard Deviation
SHY: Sleep hyperhydrosis
SWTD: Sleep-Wake Transition Disorder
TS: Total Score
